# The miR-143/-145 cluster regulates plasminogen activator inhibitor-1 in bladder cancer

**DOI:** 10.1038/bjc.2011.520

**Published:** 2011-11-22

**Authors:** S B Villadsen, J B Bramsen, M S Ostenfeld, E D Wiklund, N Fristrup, S Gao, T B Hansen, T I Jensen, M Borre, T F Ørntoft, L Dyrskjøt, J Kjems

**Affiliations:** 1 Department of Molecular Biology, Aarhus University, C.F. Møllers Allé Build, 1130, Aarhus C 8000, Denmark; 2 Department of Molecular Medicine, Aarhus University Hospital, Brendstrupgårdsvej 100, Aarhus N 8200, Denmark; 3 Department of Urology, Aarhus University Hospital, Brendstrupgårdsvej 100, Aarhus N 8200, Denmark

**Keywords:** bladder cancer, PAI-1, microRNA, miR-143, miR-145

## Abstract

**Background:**

Upregulation of the proto-oncogene plasminogen activator inhibitor-1 (PAI-1) is a common hallmark of various solid tumours, but the mechanisms controlling its expression are not fully understood.

**Methods:**

We investigate microRNAs (miRNAs) regulating PAI-1 in a panel of normal bladder urothelial biopsies, superficial Ta bladder tumours and invasive T1–T4 tumours using expression microarrays and qRT–PCR. The prognostic implications of PAI-1 deregulation are established by tissue microarray staining of non-muscle-invasive bladder tumours. MicroRNA repression of PAI-1 is assayed by ectopic miRNA expression, argonaute immunoprecipitation and luciferase assays.

**Results:**

We found that the miR-143/-145 cluster is downregulated in all stages of bladder cancer and inversely correlated with PAI-1 expression. Mature miR-143 and miR-145 are coordinately expressed, and both directly target the PAI-1 3′UTR, leading to reduced PAI-1 mRNA and protein levels. Furthermore, we show that PAI-1 and miR-145 levels may serve as useful prognostic markers for non-muscle-invasive bladder tumours for which accurate progressive outcome is currently difficult to predict.

**Conclusion:**

This report provides the first evidence for direct miRNA regulation of PAI-1 in bladder cancer. We also demonstrate mRNA co-targeting by a cluster of non-family miRNAs, and suggest miR-145 and PAI-1 as clinically relevant biomarkers in bladder cancer.

The enzyme plasminogen activator inhibitor-1 (PAI-1; encoded by the *SERPINE1* locus) is an important member of the plasminogen activator system, and is involved in various processes including thrombosis, wound healing, angiogenesis and degradation of extracellular matrix components (reviewed by Lijnen (2005)). Plasminogen activator inhibitor-1 protein levels are commonly elevated in cancers, where it is secreted into the extracellular microenvironment by tumour cells, stromal fibroblasts and tumour-associated endothelial cells (McMahon and Kwaan, 2008; Kwaan and McMahon, 2009). High PAI-1 levels in tumours usually coincide with poor prognosis, and serve as an important prognostic marker in many cancer types. Moreover, PAI-1 is known to promote cancer cell proliferation and angiogenesis by inhibiting apoptosis in cell culture systems (Kwaan *et al*, 2000; Chen *et al*, 2004). Thus, PAI-1 acts broadly as an oncogene, and further insight into its regulation will be important for future diagnostic and therapeutic developments.

MicroRNAs (miRNAs) are a class of ca. 22 nt non-coding RNAs that regulate gene expression at the level of mRNA stability and translation (Bartel, 2004). It has been estimated that miRNAs regulate the expression of 30% of protein-coding genes, and it is generally accepted that they are at some level involved in practically all cellular processes (Lewis *et al*, 2005). Many miRNAs are highly tissue specific and important for cell development and differentiation, and aberrant miRNA expression can lead to cellular dedifferentiation, oncogenesis, cancer metastasis and tumour invasion (Esquela-Kerscher and Slack, 2006). As such, widespread miRNA deregulation is a common hallmark of cancer (Wang and Lee, 2009), making miRNAs interesting as diagnostic and therapeutic targets. MicroRNA-directed deregulation of cancer-associated genes constitutes a rapidly growing research field, and decoding the underlying regulatory pathways is essential for the understanding of cancer aetiology and progression.

Both MiR-143 and miR-145 are frequently downregulated in a broad range of cancer cell lines and tumours deriving from the haematopoietic system, breast, lung, colon, prostate, the gastrointestinal system, ovary, cervix, head and neck, and bladder tissue (Volinia *et al*, 2006; Dyrskjot *et al*, 2009; Wang and Lee, 2009). These miRNAs are transcribed from a putative cluster on chromosome 5, and they are coordinately expressed in a variety of cell types and tissues (Iio *et al*, 2010). Both MiR-143 and -145 are important in maintaining the differentiated state of smooth muscle cells (SMCs), and loss of expression in SMCs correlates with vascular disease (Cordes *et al*, 2009; Elia *et al*, 2009). Moreover, ectopic expression of miR-143 and -145 in a variety of cancer cell lines leads to decreased viability (Borralho *et al*, 2009; Chiyomaru *et al*, 2010; Ostenfeld *et al*, 2010; Spizzo *et al*, 2010; Zhang *et al*, 2010a; Noguchi *et al*, 2011). Accordingly, miR-143 and miR-145 are considered to act as broad tumour suppressors, and their deregulation has been reported as an early event in transformation (Michael *et al*, 2003; Sempere *et al*, 2007).

In this study, we investigated putative miRNA regulation of mRNAs in a panel of normal bladder urothelial biopsies, superficial Ta bladder tumours and invasive T1-T4 tumours. Previously, we profiled miRNA expression in clinical bladder cancer specimens and found miR-145 to be the most downregulated miRNA in bladder tumours compared with normal urothelium (Dyrskjot *et al*, 2009). Here, we report that miR-143 and miR-145 are coordinately downregulated as an early neoplastic event in bladder cancer, which is inversely correlated with PAI-1 expression. Conversely, ectopic miR-143/-145 administration in bladder cell lines represses *PAI-1* expression by directly targeting complementary miR-143 and miR-145 sequences in the PAI-1 3′UTR. The clinical significance of PAI-1 and miR-145 expression was investigated in non-muscle-invasive tumours using immunohistochemical and *in situ* hybridisation analyses on tissue microarrays (TMAs). Accordingly, both were usable markers with elevated levels correlating with poor and good prognosis, respectively. This is the first report of PAI-1 as a direct target of miR-143 and -145, and, to our knowledge, the first demonstration of a cluster of non-family miRNAs targeting the same mRNA.

## Materials and methods

### Patient material

Bladder tumours were collected directly from surgery and processed as previously described at Aarhus University Hospital, Denmark (patient details listed in Supplementary Table 1; Dyrskjot *et al*, 2005, 2009). Tumours were graded according to the Bergkvist classification system (Carbin *et al*, 1991). Normal bladder urothelium was laser microdissected from the patients with no bladder cancer history as previously published (Dyrskjot *et al*, 2005, 2009). Informed written consent was obtained from all patients, and research protocols were approved by the local scientific ethical committee.

### Tissue microarrays

A single biopsy (0.6 mm cores) from each tumour was taken from the area chosen by an uropathologist and placed in the paraffin block using a custom-made precision instrument (manuel tissue microarrayer 1, Beecher Instruments, Sun Prairie, WI, USA). Procedures were carried out according to an already developed and published method (Kononen *et al*, 1998), and the patient cohort used for TMA construction is described by Egerod *et al* (2009). The TMA consisted of 289 primary urothelial tumours (stage Ta and T1) from patients undergoing primary TURB between 1979 and 2007. Only patients with a minimum of 4-year follow-up without progression were included in the non-progressing group. Consequently, this TMA does not represent a consecutive selected patient cohort. Patient follow-up time was from the first diagnostic resection to the most recent cystoscopy. Progression was defined as progression to muscle-invasive disease and was verified by pathological examination in all patients. Patients who died before progression were censured as uneventful at the time of death. Patients undergoing cystectomy before progression to muscle-invasive disease were not included on the TMA.

### Cell culture and transfections

All bladder cell lines were supplied by Professor Pamela J. Russell (Prince of Wales Hospital, Sydney, Australia). All other cell lines were available at the Department of Molecular Biology, Aarhus University, Aarhus, Denmark. Except for the H1299 cell line, all cells were propagated in Dulbecco's modified Eagle's medium supplemented with 10% fetal calf serum (Invitrogen, Carlsbad, CA, USA), 50 U ml^−1^ penicillin and 50 mg ml^−1^ streptomycin (both from Sigma-Aldrich (St Louis, MO, USA) under standard conditions (37 °C, 5% CO_2_).

H1299 cells were maintained in Roswell Park Memorial Institute Medium 1640 supplemented and in conditions as for the other cell lines.

All transfections were carried out using Lipofectamine 2000 (Invitrogen), according to manufacturers protocol.

All miRNAs and the Scr. Ctrl. used for transfections were Pre-miR miRNA Precursor Molecules and Negative Control, respectively (Applied Biosystems, Foster City, CA, USA). The Scr. siRNA control is targeting the sequence 5′-GACGUAAACGGCCACAAGU-3′ of non-endogenous eGFP (Bramsen *et al*, 2009). All miRNAs were transfected in a final concentration of 50 nM, 25 nM when in combination with another miRNA and all siRNAs were transfected in a 25 nM final concentration.

### Microarray analyses

RNA was extracted using Trizol Reagent (Invitrogen) and quality was controlled using a 2100 Bioanalyzer (Agilent Technologies, Santa Clara, CA, USA). For gene expression, the RNA was labelled and hybridised to Human Exon 1.0 ST Arrays (Affymetrix, Santa Clara, CA, USA) and analysed as previously described (Thorsen *et al*, 2008). MicroRNA expression analysis was made using the miRCURY LNA Array Ready to Spot v. 7.1 assay (Exiqon, Vedbaek, Denmark) and analysed as previously described (Schepeler *et al*, 2008).

### *In situ* hybridisation

The miR-145 *in situ* hybridisation was generated and evaluated in a previous study (Ostenfeld *et al*, 2010). The miR-145 LNA probe had the sequence: 5′-AGGGATTCCTGGGAAAACTGGAC-3′. To verify probe specificity, a probe with two mismatched nucleotides (underlined, bold) was also used: 5′-AGGGATTCCTGGGAAAA**G**T**C**GAC-3′. Staining pictures were made using an Olympus IX71 inverted microscope and the cellSens Digital Imaging Software (both from Olympus, Tokyo, Japan).

### Immunohistochemical staining

Immunohistochemistry was performed on formalin-fixed paraffin embedded 4-*μ*m tissue sections transferred to Menzel Superfrost-Plus slides. The TMA slide was incubated with the primary antibody for 60 min at room temperature. The DAKO EnVision+ visualisation system (DAKO, Glostrup, Denmark) was used to visualise the antigen through a chromogen reaction. Immunohistochemistry was performed essentially as described by Mansilla *et al* (2007) using rabbit polyclonal antibodies to PAI-1 (Offersen *et al*, 2003). Staining (yes/no) of each biopsy on the TMA was scored blinded to clinical outcome.

### Gene expression analyses

RNA was purified from cells using the Trizol Reagent (Invitrogen), according to manufacturers protocol. First strand cDNA was synthesised using MLV Reverse Transcriptase (Invitrogen) with random hexamer priming from 1 *μ*g of total RNA starting material. The reactions were diluted 10 times, and 4 *μ*l cDNA was used per 10 *μ*l PCR amplification. Gene expression was analysed using Platinum SYBR Green qPCR SuperMix-UDG (Invitrogen) on a LightCycler 480 (Roche, Basel, Switzerland; 40 cycles, 62°C annealing). Intron-spanning primers were used for all genes:

PAI-1 F: 5′-GAGGTGCCTCTCTCTGCCCTCACCAACATT-3′

PAI-1 R: 5′-AGCCTGAAACTGTCTGAACATGTCG-3′

GAPDH F: 5′-GTCAGCCGCATCTTCTTTTG-3′

GAPDH R: 5′-GCGCCCAATACGACCAAATC-3′.

Expression was normalised to GAPDH using the 2^−ΔCt^ method.

### miRNA expression analyses

RNA was purified from cells using the Trizol Reagent (Invitrogen), according to manufacturers protocol. MicroRNA expression in cell lines was determined by miRNA TaqMan qRT–PCR assays (Applied Biosystems) on a LightCycler 480 (Roche) according to supplier's protocol. Expression was normalised to RNU48 or RNU43 TaqMan assays as indicated using the 2^−ΔCt^ method.

### Western blotting

Lysis of cells was carried out using a freshly made RSB300 buffer (100 mM Tris–HCl (pH 7.4), 300 mM NaCl, 2.5 mM MgCl_2_, 40 *μ*g ml^−1^ digitonin, 0.5% Triton X-100 (all from Sigma-Aldrich), Complete Mini protease inhibitor tablets (Roche). Protein fractions were normalised using the Bradford method as previously described (Bradford, 1976) and loaded on denaturing 12% SDS–PAGE gels, electroblotting was performed on Immobilon-P Membranes (Millipore, Billerica, MA, USA) O/N, and a primary rabbit anti-human-PAI-1 antibody (Offersen *et al*, 2003) was applied followed by a commercial HRP-conjugated Polyclonal Goat Anti-Rabbit Antibody (DAKO) and visualisation using SuperSignal West Femto Chemiluminescent Substrate (Rockford, IL, USA).

### Argonaute immunoprecipitation

Fifty percent confluent HeLa cells in p10 plates were transfected with Pre-miR miRNA Precursor Molecules (Applied Biosystems), pFLAG-Ago1 and pFLAG-Ago2 (pFLAG-Ago1/2 purchased from Addgene, Cambridge, MA, USA) using Lipofectamine 2000 (Invitrogen) and harvested 48 h later. Immunoprecipitation was performed as previously described (Hendrickson *et al*, 2008) employing anti-FLAG antibody-conjugated agarose beads (Sigma-Aldrich). Samples were DNAse treated using DNA free (Ambion, Foster City, CA, USA) and random hexamer primed reverse transcription of RNA from IPs, and inputs were subsequently performed using MLV Reverse Transcriptase (Invitrogen) according to manufacturers protocol.

Quantitative reverse transcriptase–PCR on pulldown and inputs was essentially made as described in ‘Gene expression analysis’ section with a further normalisation to IP/input of GAPDH using the 2^−ΔCt^ method.

### Construction of luciferase reporter cell lines and usage in assay

A partial *PAI-1* 3′UTR sequence of 700 bp containing the miR-145 and -143 target sites were cloned into psiCheck2-PURO using the *Xho*I and *Not*I restriction sites –a modified psiCheck2 vector (Promega, Madison, WI, USA) containing a puromycin-resistance gene (as described by Bramsen *et al* (2010)). The plasmid generated was named psiCheck2-PURO-trunc PAI-1 3′UTR wt.

Moreover, a miR-143/-145 target-site double-mutant construct of the plasmid described above was generated using an overlap PCR approach. This plasmid was named psiCheck2-PURO-trunc *PAI-1* 3′UTR target-site db. mut.

In addition, two constructs with the isolated *PAI-1* 3′UTR miR-143 and miR-145 target sites, respectively, were made by annealing 50-mer DNA oligos with *Xho*I/*Not*I overhangs and ligating these into psiCheck2-PURO. Furthermore, mutants of these were made.

Primers used for plasmid constructions:

Outer PAI-1 F 5′-CTACCTCGAGAGTCCTTTTCATGGGCCAAGT-3′

Outer PAI-1 R 5′-GCATGCGGCCGCAAAATAACCCATGCACACTGTTTCT-3′

miR-145 TS mut F 5′-ATCTGGGACAATTGCTCTGATGCATCGGG-3′

miR-145 TS mut R 5′-CCCGATGCATCAGAGCAATTGTCCCAGAT-3′

miR-143 TS mut F 5′-GAAACACCCTTAGTAGATGGAGTCCACTGTG-3′

miR-143 TS mut R 5′-CACAGTGGACTCCATCTACTAAGGGTGTTTC-3′

miR-145 seed wt S 5′-ACGTCTCGAGTCATCTGGGACAAAACTGGAGATGCATCGCGGCCGCCTAG-3′

miR-145 seed wt AS 5′-CTAGGCGGCCGCGATGCATCTCCAGTTTTGTCCCAGATGACTCGAGACGT-3′

miR-145 seed mut S 5′-ACGTCTCGAGTCATCTGGGACAACGATCGTGATGCATCGCGGCCGCCTAG-3′

miR-145 seed mut AS 5′-CTAGGCGGCCGCGATGCATCACGATCGTTGTCCCAGATGACTCGAGACGT-3′

miR-143 seed wt S 5′-ACGTCTCGAGGAAGAAACACCCTTTCATCTCAGAGTCCGCGGCCGCCTAG-3′

miR-143 seed wt AS 5′-CTAGGCGGCCGCGGACTCTGAGATGAAAGGGTGTTTCTTCCTCGAGACGT-3′

miR-143 seed mut S 5′-ACGTCTCGAGGAAGAAACACCCTTCGATCGGTGAGTCCGCGGCCGCCTAG-3′

miR-143 seed mut AS 5′-CTAGGCGGCCGCGGACTCACCGATCGAAGGGTGTTTCTTCCTCGAGACGT-3′

Stable luciferase reporter H1299 cell lines were generated as described previously (Bramsen *et al*, 2010). Cells were reverse transfected in biological sextuplicates into 96-well plates (5000 cells, 0.5 *μ*l Lipofectamine 2000 per well) and luciferase activity was assayed 30 h later using the Dual-Luciferase Reporter Assay System (Promega) and the FLUOstar Optima (BMG Labtech, Ortenberg, Germany).

### Statistical analyses

Generally, statistical significance was determined by 2-tailed Student's *t*-test or *χ*^2^-test (as indicated). Correlation *P*-value of miR-143 and -145 from microarray analysis was calculated using the ANOVA regression analysis. These analyses as well as Pearson correlation factors were calculated using Microsoft Excel 2004 for Macintosh. We used Stata 10.0 statistical analysis software (Stata Corporation, College Station, TX, USA) for calculation of log-rank tests for equality of survival function and for the generation of Kaplan–Meier survival estimates. The assumptions of proportional hazards were verified.

## Results

### The miR-143/-145 cluster and PAI-1 are deregulated in bladder cancer samples

**Figure 1:**
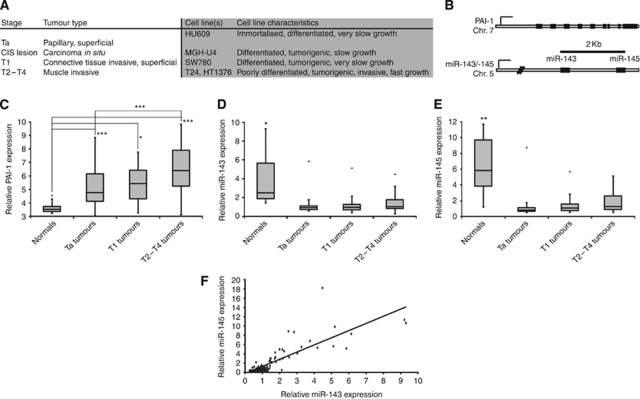
PAI-1 and miR-143/-145 expression in clinical bladder specimens. (**A**) White background: overview of bladder cancer stage classification. Grey background: description of cell lines used in this study placed next to tumour grade origin (adapted from Wiklund *et al* (2011)). (**B**) Graphical depiction of the *PAI-1* and *miR-143/-145* genomic loci. (**C**) Affymetrix exon array relative PAI-1 expression in normal bladder tissues (*n*=9), Ta tumours (*n*=49), T1 tumours (*n*=8) and T2–T4 tumours (*n*=34). (**D** and **E**) Exiqon LNA array relative expression of miR-143 and -145 in normal bladder tissues (*n*=11), Ta tumours (*n*=30), T1 tumours (*n*=47) and T2-T4 tumours (*n*=27; adapted from Dyrskjot *et al* (2009)). (**F**) Scatter plot analysis of miR-143/-145 expression correlation in the bladder samples from (**D** and **E**; *P*=5.6E-33). (^*^P<0.05, ^**^P<0.01, ^***^P<0.001 compared with all other groups individually or as indicated with bridging bars. Rounded dots in box plots indicate maximum outliers. *P*-values determined by 2-tailed Student's *t*-test).

Profiles of mRNA and miRNA steady-state levels were assayed in tissue samples from normal bladder urothelial biopsies, superficial Ta bladder tumours and invasive T1–T4 tumours (Figure 1A) using Affymetrix Exon ST 1.0 microarrays and Exiqon LNA-based microarrays, respectively, and expression of PAI-1 and the clustered miR-143/-145 (Figure 1B) were investigated in detail. Plasminogen activator inhibitor-1 mRNA levels were upregulated from the less invasive tumour stages and onwards (Figure 1C), whereas miR-143 and -145 were among the most downregulated miRNAs in bladder tumours compared with normal urothelium (Dyrskjot *et al*, 2009), and expressed only at background levels in all stages of bladder cancer (Figures 1 D and E). Overall, PAI-1 expression levels were highly variable in the bladder cancer samples, while miR-143 and -145 were downregulated to a similar extent in all tumours.

To confirm that miR-143 and miR-145 are co-expressed and deregulated as a cluster in bladder cancer (Figure 1B), scatter plot analysis was performed to compare relative expression in the clinical samples (Figure 1F). Indeed, the expression appeared tightly coordinated with a Pearson correlation factor of 0.83 (ANOVA regression *P*=5.6E-33) strongly suggesting that miR-143 and -145 are transcribed as a cluster.

### PAI-1 and miR-145 are potential prognostic markers in bladder cancer

**Figure 2:**
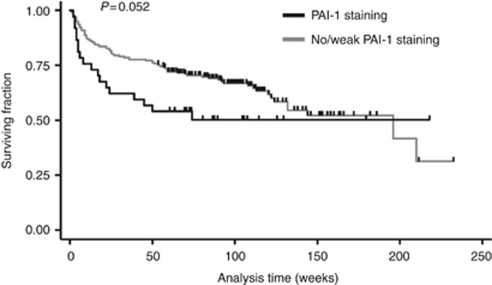
PAI-1 as a prognostic marker in non-muscle-invasive bladder tumours. Plasminogen activator inhibitor-1 Kaplan–Meier survival plot determined for 258 patients with Ta tumours (*n*=164) and T1 tumours (*n*=94; *P*=0.052).

Next, we used immunohistochemical staining and LNA-based *in situ* hybridisation on TMAs to assess the clinical significance of PAI-1 protein and mature miR-145 levels. Stained tissue sections from 164 Ta and 94 T1 tumours were scored blinded and compared with disease outcome (Figure 2). Plasminogen activator inhibitor-1 expression was positively correlated with poor prognosis in all patients (*P*=0.052) and, as previously published, positive miR-145 staining correlated with a favourable disease outcome for patients with T1 tumours (*P*=0.057; Ostenfeld *et al*, 2010). *In situ* staining of MiR-143 was not performed because its expression is directly coupled to miR-145, as described above. In all the tumours, PAI-1 and miR-145 localised predominantly to the cytoplasm; localisation of the latter being a general prerequisite for repression potential (Bartel, 2004; Supplementary Figure 1). This suggests PAI-1 levels as a potential prognostic marker in all superficial stages of urothelial carcinoma, while the loss of miR-143/-145 expression in the first step of invasion is indicative of poor disease outcome.

### MiR-143/-145 and PAI-1 expression are inversely correlated in bladder cancer

**Figure 3:**
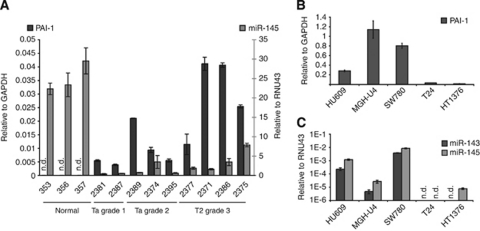
Correlation of PAI-1 and miR-143/-145 expression in clinical bladder samples and cell lines assayed by qRT–PCR. (**A**) Matched PAI-1 and miR-145 expression in nine bladder cancer tumours (five Ta tumours, four T2 tumours) and in normal bladder urothelium from three healthy volunteers. (**B**) Plasminogen activator inhibitor-1 expression in a panel of four bladder cancer cell lines (MGH-U4, SW780, T24 and HT1376) and a normal immortalised urothelial cell line (HU609). (**C**) Expression of miR-143 and miR-145 in the same cell line panel as above. Abbreviation: n.d., not detectable.

Our initial expression screen pointed to a dynamic inverse correlation between miR-143/-145 and PAI-1 expression during the development of bladder cancer (Figure 1). To confirm this observation, we compared matched mRNA/miRNA sample sets from the microarrays. A negative correlation between PAI-1 and both the miRNAs was observed when comparing miRNA and mRNA levels in 27 patients (Pearson correlation factor −0.28 and −0.35 between PAI-1 *vs* miR-143 and miR-145, respectively). Furthermore, we methodically substantiated this finding using qRT–PCR on 12 representative samples, from which we had sufficient RNA material left from microarray analysis. These were nine patient samples (six Ta tumours and three T2 tumours) and bladder mucosa biopsies from three healthy individuals. A clear inverse correlation between miRNA and mRNA expression was observed (Pearson correlation factor −0.44; Figure 3A). In accordance with the microarray data, reduction of miR-145 expression was apparent already in superficial carcinomas, with Ta tumours showing the lowest steady-state levels. The increase in PAI-1 expression is evident in all cancer stages, thus, potentially providing a diagnostic marker for tumour invasion.

Next, endogenous levels of PAI-1 mRNA and miR-143/-145 were investigated in a panel of bladder cell lines (see Figure 1A for cell line descriptions). Consistent with the clinical data, we observed a higher expression of PAI-1 in cell lines isolated from superficial tumours (SW780 and MGH-U4) compared with immortalised urothelial cells, (HU609; Figure 3B). In HU609 cells and in the two relatively well-differentiated cell lines, SW780 and MGH-U4, PAI-1 is highly expressed with the HU609 cell line having the lowest expression (Figure 3B). In comparison, steady-state levels of PAI-1 are low in the more advanced, poorly differentiated cancer cell lines T24 and HT1376. Furthermore, miR-143 and -145 expression levels in the cell line panel follow a similar pattern as the clinical samples, with miRNA levels decreasing to background levels in the undifferentiated cell lines (Figure 3C). Notably, miR-143/-145 and PAI-1 levels in the three most differentiated cell lines are inversely represented with MGH-U4, having the highest PAI-1 and the lowest miR-143/-145 expression. Thus, the direct comparison of miRNA and mRNA levels in tissue samples, and to some degree in cell lines, confirm that expression of PAI-1 and miR-143/-145 are mutually exclusive.

### Ectopic miR-143 and miR-145 expression reduce endogenous PAI-1 mRNA and protein levels

**Figure 4:**
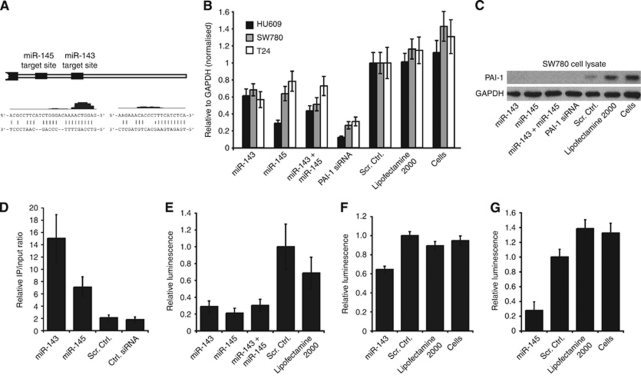
Effect of miR-143/-145 transfection on PAI-1 levels in bladder cell lines and identification of miRNA target sites. (**A**) Upper part: depiction of the miR-143 and -145 target sites in the proximal part of the PAI-1 3′UTR. Lower part: schematic representation of the putative miR-145 (left) and miR-143 (right) binding-site interactions with the 3′UTR and the conservation of each target site. (**B**) PAI-1 expression assayed by qRT–PCR following 24 h transient transfection of three bladder cell lines with the miRNA mimics/siRNA indicated. (**C**) PAI-1 protein levels in SW780 cell lysates from the experiment in the previous figure panel. (**D**) Argonaute IP and PAI-1 qRT–PCR on RNA from HeLa cells transfected with plasmids expressing tagged argonaute 1/2 and the miRNAs/siRNAs indicated. (**E**) Luciferase assay showing the miRNA effects on cells stably expressing a reporter with a large part of the PAI-1 3′UTR normalised to a similar reporter cell line with mutated target sites for miR-143 and -145. (**F**) Luciferase assay with cell lines expressing truncated isolated miR-143 target-site reporters. Again, the wild-type target reporter cell line is normalised to one with a mutated target site. (**G**) As in (**F**), but with a miR-145 target-site reporter normalised to a target-site mutant.

The inverse expression pattern of miR-143/-145 and PAI-1 prompted us to consider a miRNA regulatory mechanism as an explanation for our observations. Using the miRNA target prediction algorithms TargetScan and RNAHybrid (Rehmsmeier *et al*, 2004; Lewis *et al*, 2005), we found that miR-143 and -145 were strong candidate regulators of PAI-1, with putative target sites located 500 bp apart in the 2-kb PAI-1 3′UTR (Figure 4A). The predicted miR-143 target is a relatively poorly conserved 8-mer site, whereas the putative 7-mer-m8 binding site for miR-145 is highly conserved and situated in the immediate proximal part of the 3′UTR (Figure 4A).

To test miR-143/-145 targeting experimentally, HU609, SW780 and T24 cell lines were transfected with combinations of precursor miRNA mimics to assess the effect on endogenous PAI-1 expression (Figure 4B). In HU609 and SW780 cells, PAI-1 mRNA levels decreased upon transfection with miR-143 and/or miR-145, indicating that both members of the miRNA cluster have a potent PAI-1 silencing potential. The effect of miR-143/-145 transfection was also assayed by western blot in the high PAI-1-expressing cell line SW780 (Figure 4C). In accordance with the mRNA destabilisation data, a dramatic PAI-1 repression was observed with both miRNAs, either individually or in combination. Plasminogen activator inhibitor-1 protein levels were also moderately reduced in scrambled control compared with non-transfected cells, which could be attributed to RNAi pathway saturation and/or secondary off-target effects affecting PAI-1 protein regulatory factors, as previously described for other cases (Grimson *et al*, 2007; Khan *et al*, 2009). However, downregulation of PAI-1 expression by miR-143 and -145 was also confirmed in other cancer cell lines, including H1299 (non-small cell lung carcinoma), HeLa (cervical cancer) and U251 (glioma cell line), thereby demonstrating that miR-143/-145-directed repression of PAI-1 is not restricted to bladder cancer (Supplementary Figure 2).

### miR-143 and -145 directly bind and repress PAI-1 through distinct 3′UTR target sites

To verify the direct association between miR-143/-145 and PAI-1, we used an argonaute immunoprecipitation assay. FLAG-tagged Ago-1, -2 and miRNA precursors were transfected into PAI-1-expressing HeLa cells, followed by Ago-IP and mRNA qRT–PCR. Plasminogen activator inhibitor-1 mRNA was significantly enriched upon miR-143 and -145 transfection compared with negative controls, suggesting direct interaction between miR-143/-145, RISC complexes and PAI-1 mRNA (Figure 4D).

To validate the target-site interaction, stable cell lines expressing luciferase reporters containing a 700-bp fragment of the *PAI-1* 3′UTR encompassing both miRNA target sites, or corresponding target-site mutants, were generated. Reporter knockdown was measured upon transfection with different combinations of miRNAs. Both individually and in combination, miR-143 and -145 caused a potent downregulation of luciferase activity compared with target-site mutants and controls (Figure 4E). To validate the isolated target sites, luciferase assays were performed using reporter constructs containing ∼50 nt fragments of the *PAI-1* 3′UTR harbouring the miR-143 and -145 target-sites and seed-mismatch mutants (Figures 4F and G). Repression of luciferase activity was only observed when a miRNA was cotransfected with the reporter construct harbouring the respective target site, thus confirming target sequence dependency.

## Discussion

Some of the most frequently repressed miRNAs in cancer include MiR-143 and -145, however, their role as broad tumour suppressors has yet to be fully understood mechanistically. Here we report that miR-143 and -145 directly repress the proto-oncogene PAI-1 in bladder cancer. The expression of PAI-1 and miR-143/145 were inversely correlated in primary bladder samples, and high PAI-1 expression and low miR-143/-145 levels correlated with poor prognosis in patients with non-muscle-invasive tumours. Moreover, we were able to map the exact target sites of the miRNAs in the PAI-1 3′UTR, thereby for the first time demonstrating a direct interaction between a cluster of non-family miRNAs and an mRNA with direct implications in cancer aetiology.

The PAI-1 mRNA is known to be stabilised by its 3′UTR, suggesting that this non-coding sequence is important for its regulation (Shetty *et al*, 2008). A recent report showed that miR-30c and -301a represses PAI-1 via 3′UTR target sites (Patel *et al*, 2011), and there is circumstantial evidence that PAI-1 is downregulated by miR-449a/-b and miR-146a (Li *et al*, 2010; Muth *et al*, 2010, 2011). In line with the direct target interaction described herein, one study reported PAI-1 mRNA silencing upon miR-145 transfection in breast cancer cells (Gotte *et al*, 2010).

In the present study, we observed a dramatic increase in PAI-1 mRNA levels in bladder cancer specimens compared with normal urothelium. Furthermore, PAI-1 expression increased with advancing tumour stage, and steady-state levels appeared more dispersed in muscle-invasive tumours. We also measured a high level of PAI-1 in bladder cancer cell lines, but expression gradually declined in more dedifferentiated lines. Concordant with our results, a significant upregulation of PAI-1 in tissue and plasma of bladder cancer patients compared with healthy individuals has previously been reported (Becker *et al*, 2010). However, no variations in PAI-1 levels has been described between invasive and superficial tumours (McGarvey *et al*, 1998; Becker *et al*, 2010). Thus, the high expression of PAI-1 in bladder cancer samples and cell lines is broadly consistent with the current literature, suggesting a selective pressure for this proto-oncogene during transformation.

In line with the PAI-1 activation observed in superficial tumours, we also found an early stage repression of the miR-143/-145 cluster without a significant difference between invasive and superficial tumours. This was further substantiated by the evident loss of miR-143 and -145 expression in dedifferentiated, invasive bladder cell lines. Also, miR-143 and -145 expression was tightly correlated in clinical specimens and cell lines, in accordance with the recent identification of a common transcription start site for these miRNAs (Kent *et al*, 2010). Silencing of the *miR-143/-145* cluster has been broadly reported in cancer, including bladder cancer (Dyrskjot *et al*, 2009; Wang and Lee, 2009). This has been attributed to transcriptional modulation, impaired processing and promoter hypermethylation (Michael *et al*, 2003; Sachdeva *et al*, 2009; Suzuki *et al*, 2009; Kent *et al*, 2010; Ostenfeld *et al*, 2010; Zaman *et al*, 2010). Accordingly, our data suggest a coordinated loss of miR-143/-145 expression as an early event in bladder carcinogenesis.

Collectively, these observations are indicative of an inverse relationship between PAI-1 and miR-143/-145 in primary samples, which is further substantiated by a direct comparison of expression levels in matched clinical samples assayed by microarray and qRT–PCR. However, this expression pattern was not identical in the bladder cell line panel where some of the most differentiated cell lines (HU609, MGH-U4 and SW780) showed the highest PAI-1 levels and lowest miR-143/-145 expression, while both miR-143/-145 and PAI-1 levels were low in the more dedifferentiated cell types (T24 and HT1376).

Plasminogen activator inhibitor-1 repression is a hallmark of several cancer cell lines, and this deregulation has been linked to several factors, including p53, TGF-*β*, and genetic and epigenetic effects (Hiti *et al*, 1990; Gao *et al*, 2005; Nagamine, 2008; Shetty *et al*, 2008). In fact, global gene silencing by promoter hypermethylation is a widespread feature of advanced tumours and dedifferentiated cell lines, and has been reported to repress PAI-1 in several cancer cell lines (Gao *et al*, 2005; Esteller, 2008). Thus, the combined low expression of miR-143/-145 and PAI-1 in dedifferentiated bladder cell lines may therefore be the result of multiple simultaneous aberrations, and the contribution of miRNA regulation could be shielded by other more dominant effects. Nonetheless, our data imply that miR-143/-145 and PAI-1 are inversely expressed in bladder tumours and differentiated bladder cell lines, suggesting that they share a common regulatory pathway.

Using a variety of approaches, we provide evidence for direct PAI-1 regulation by miR-143 and -145 through a non-conserved 8-mer target site and a conserved 7-mer target site, respectively. Notably, repression of endogenous PAI-1 and PAI-1 3′ luciferase reporters was more potent for miR-145 than for miR-143. In contrast, the enrichment of PAI-1/miRNA/RISC complexes was higher in miR-143- as compared with miR-145-transfected cells. MicroRNA target site conservation is a known determinant of miRNA function, and current evidence suggests that repression activity is more potent for 8-mer compared with 7-mer target sites (Lewis *et al*, 2005; Friedman *et al*, 2009; Lee *et al*, 2011). Although both miRNAs are functional PAI-1 regulators, miR-145 appear more potent, thus, indicating that conservation is a more important factor than target site length for determining PAI-1 miRNA repression potency. Interestingly, the coordinated repression capability of PAI-1 by miR-143 and 145 is an immediate consequence of the two spatially distinct target sites. As such, this repression is tightly controlled, and several mutations are required to obstruct the functional miRNA regulation of PAI-1. A similar phenomenon may also be involved in the regulation of CUGBP2 in cardiomyocytes by the miR-144/-451 cluster, but this target needs to be validated and functionally substantiated by expression of ectopic miRNAs (Zhang *et al*, 2010b).

Tissue microarray analysis of non-muscle-invasive Ta and T1 tumours suggest that expression of PAI-1 and the miR-143/-145 cluster are suitable predictors of disease outcome. A previous study showed that PAI-1 expression in invasive T2–T4 tumours correlates with poor prognosis bladder cancer (Becker *et al*, 2010). Here, we validate PAI-1 expression in a large number of stage Ta/T1 tumours, demonstrating that PAI-1 is a predictor of aggressive progression already at an early invasive stage. Reduced expression of the miR-143/-145 cluster, as determined by miR-145 *in situ* hybridisation in T1 tumours, is also a predictor of poor prognosis (Ostenfeld *et al*, 2010). A critical obstacle in bladder cancer treatment is to characterise the carcinogenic potential of Ta and T1 tumours for which it is intrinsically difficult to predict disease outcome (Fujikawa *et al*, 2003). Thus, PAI-1 and miR-143/-145 deregulation have potential clinical significance for early diagnosis and treatment assessment of bladder cancer.

In conclusion, we find that PAI-1 is coordinately targeted by a cluster of non-family miRNAs in bladder cancer. To our knowledge, the regulation of PAI-1 by miR-143/145 is the first reported example of a multi-seed combinatorial miRNA mechanism deregulating an oncogene. This is also the first description of three mechanistically linked molecules with prognostic potential for non-invasive bladder tumours.
