# Supplementary figures and images for: The miR-143/-145 cluster regulates plasminogen activator inhibitor-1 in bladder cancer
Source: Br J Cancer. 2011 Nov 22;106(2):366–74. doi: 10.1038/bjc.2011.520 (PMC3261682; doi:10.1038/bjc.2011.520)

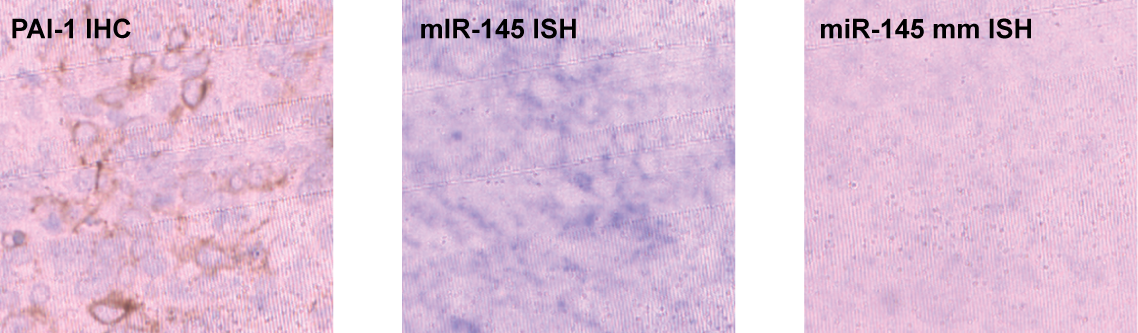

Supplement: Supplementary Figure 1 [file bjc2011520x1.png]

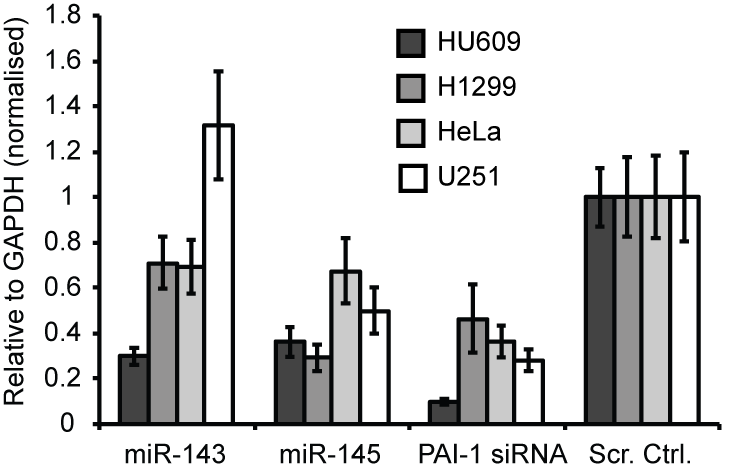

Supplement: Supplementary Figure 2 [file bjc2011520x2.png]
