# Supplementary material for: The miR-143/-145 cluster regulates plasminogen activator inhibitor-1 in bladder cancer
Source: Br J Cancer. 2011 Nov 22;106(2):366–74. doi: 10.1038/bjc.2011.520 (PMC3261682; doi:10.1038/bjc.2011.520)
Supplement: Supplementary Legends [file bjc2011520x4.doc]

**Supplementary table 1: Patient data.** Details on patients analysed for mRNA and/or miRNA expression as indicated.

**Supplementary figure 1: Tissue microarray example.** Tissue microarray biopsy stained for PAI-1, miR-145 and a miR-145 mismatch (mm) control.

**Supplementary figure 2: Effect of ectopic miR-143/-145 administration on PAI-1 levels in cell lines of various origins.** PAI-1 expression assayed by qRT-PCR 48 hours after transfection with the miRNAs/siRNAs indicated.
